# Supply and Demand Determine the Market Value of Access to Infants in the Golden Snub-Nosed Monkey (*Rhinopithecus roxellana*)

**DOI:** 10.1371/journal.pone.0065962

**Published:** 2013-06-12

**Authors:** Wei Wei, XiaoGuang Qi, Paul A. Garber, SongTao Guo, Pei Zhang, BaoGuo Li

**Affiliations:** 1 Key Laboratory of Resource Biology and Biotechnology in Western China, College of Life Sciences, Northwest University, Xi’an, China; 2 Institute of Zoology, Shaanxi Academy of Sciences, Xi’an, China; 3 Anthropology Department, University of Illinois, Urbana, Illinois, United States of America; Université de Strasbourg, France

## Abstract

According to a biological market paradigm, trading decisions between partners will be influenced by the current ‘exchange rate’ of commodities (good and services), which is affected by supply and demand, and the trader’s ability to outbid competitors. In several species of nonhuman primates, newborn infants are attractive to female group members and may become a desired commodity that can be traded for grooming within a biological market place. We investigated whether grooming was interchanged for infant handling in female golden snub-nosed monkeys (*Rhinopithecus roxellana*) inhabiting the Qinling Mountains of central China. *R. roxellana* exhibit a multilevel social organization characterized by over 100 troop members organized into 6–11 one-male units each composed one adult male and several adult females and their offspring. Behavioral data were collected over the course of 28 months on grooming patterns between mothers with infants less than 6 months old (*N* = 36) and other adult female troop members. Our results provide strong evidence for the interchange of grooming for access to infants. Grooming for infant access was more likely to be initiated by potential handlers (nonmothers) and less likely reciprocated by mothers. Moreover, grooming bout duration was inversely related to the number of infants per female present in each one-male unit indicating the possibility of a supply and demand market effect. The rank difference between mothers and handlers was negatively correlated with grooming duration. With increasing infant age, the duration of grooming provided by handlers was shorter suggesting that the ‘value’ of older infants had decreased. Finally, frequent grooming partners were allowed to handle and maintain access to infants longer than infrequent groomers. These results support the contention that grooming and infant handling may be traded in *R. roxellana* and that the price individuals paid for access to infants fluctuated with supply and demand.

## Introduction

Biological market theory [1], [2] offers a set of predictions regarding dynamic changes in the value and exchange of goods and services among members of a social group. This explanatory framework offers a new perspective on the costs and benefits of cooperative behavior under conditions in which individuals have the opportunity to interact with a diverse set of partners, and the different individuals within a given trader class compete to outbid each other for access to valued commodities [3]. The principles of a biological market reflect the natural interactions of group-living animals, in which potential cooperators differ in the quality or type of services they can offer in exchange for the quality and type of services they require [1], [2]. In this regard, two primary trader classes have been described: ‘sellers’, provide a specific commodities, and ‘buyers’, choosing the most valuable commodity. [1], [2]. Potential cooperators also may differ in their access to or preference for particular trading partners based on age, dominance status, kinship, and previous social histories. Within the ‘biological market’ paradigm, traders are expected to seek partners who offer the optimal commodity value based on current supply and demand as well as their ability to effectively outbid competitors for these goods and services [2]. Scarcity in terms of the availability of goods, services, or particular traders (i.e. access to a high ranking coalitionary partner) and increased individual need, should lead to an increase in the cost of obtaining that commodity, whereas under conditions of surplus there is an expected reduction in cost [1], [2].

Grooming, is the most common affiliative behavior engaged in by nonhuman primates [4], [5]. And, although grooming is likely to serve several social functions, it plays a primary role in establishing and reinforcing social bonds [6], [7], [8] and in reducing social tension and promoting reconciliation between group mates [9], [10], [11].Therefore, grooming represents a beneficial service that primates may exchange or interchange for other commodities [7], [12], [13].

Previous research has demonstrated that primate ‘reciprocal traders’ commonly exchange or reciprocate grooming for grooming [14], [15], [16], [17], [18], [19], [20]. In addition, grooming may be swapped for other commodities or services among ‘interchange traders’. Across primate species, grooming has been interchanged for coalitionary support [21], food [22], [23], tolerance [24], mating opportunities [25], [26], [27], food sharing [28] and access to information concerning female reproductive status [19], [29].

In several species of primates, newborn infants are very attractive to group members, especially females, and mothers with young infants receive high levels of social attention [13], [30], [31]. Females often groom mothers in an attempt to touch, handle, nuzzle and inspect their infants [32], [33]. Maternal restrictiveness and the attraction that females have for infants make them a valuable and restricted commodity [34], [35]. Moreover, given that the postnatal costs of infant care (nursing, transporting, food sharing, and protecting infants over a relatively extended developmental period) for female primates can be high [30], mothers in many primate species are tolerant of aunts or helpers who contribute to infant care [36], [37], [38], [39], [40]. Using a biological market framework, females without infants may be expected to interchange grooming or other service for access to infants (hereafter handlers who may gain critical infant care experience that increases the survivorship of their future offspring or develop strong social bonds with the infant’s mother). This creates a demand for access to infants [41], [42], [43], [44]. Henzi & Barrett (2002) [41] provided the first test of the biological market model on grooming and infant handling in the context of interchange trading. In the case of chacma baboons (*Papio hamadryas ursinus*) these authors found that mothers were groomed for longer periods of time when there were fewer newborns in the group, a finding confirmed in several primate studies [42], [43], [45], but not in others [44], [46]. In the present study, we use a biological market framework to examine social interactions, grooming relationships, and access to infants in female golden snub-nosed monkeys (*Rhinopithecus roxellana*). Observations of this species indicate that females without infants are attracted to females with young infants and groom them frequently [13].

The golden snub-nosed monkey (*Rhinopithecus roxellana*) is a rare and endangered colobine primate endemic to China. Colobines are foregut fermenters and possess adaptations of their stomach (multichambered, enlarged, low PH, highly diverse microbacterial biome) that enable them to exploit a diet composed primarily of leaves, lichen, seeds, and other difficult to digest plant material [39], [47]. The social organization of *R. roxellana* is characterized by a multi-level society [48] composed of several one-male units (OMUs) and an associated all-male band. Troop size commonly exceeds 100 individuals [49], [50]. The OMU is the basic social and reproductive unit and consists of a single resident male, 3–7adult females, subadults, juveniles, and infants [13], [48], [51]. Troop size is reported to fluctuate across seasons, with a decrease in the number of one-male units that foraging together during periods of low food availability (November-nest year January). Males leave their natal OMU before sexual maturity and join an all-male band. Although some females remain in their natal OMU and breed, other females disperse into different OMUs within the troop or on occasion into a new troop [52]. *R. roxellana* is a strictly seasonal breeder [53]. The mating season is from September to December, and the birth season is from March to May [54]. Gestation is approximately 6–7 months, and females with surviving offspring give birth once every two years. Given that the average number of adult females per OMU is 4.76±1.57 (mean ± SD) and females breed once every two years, the number of infants (<6 months old) in each OMU is approximately 1.8±1.3 (mean ± SD).

Female golden snub-nosed monkeys without infants are attracted to newborn infants. Access to young infants requires than that nonmothers cautiously approach mothers and either groom them or sit near them [13]. In the biological market place that is the golden snub-nosed monkey OMU, access to infants is a commodity desired by females without infants, and therefore, differences in rank between mother and handler, as well as infant age, are expected to influence the ‘value’ of goods and services interchanged during social interactions. In order to examine these relationships more clearly, we test the following predictions:

1. Grooming sessions between nonmothers and mothers are primarily unidirectional and nonreciprocal. Nonmothers are expected to groom mothers for a longer duration than they groom other females.
2. Grooming as a means of ‘purchasing’ access to infants is less likely to occur between mother–mother dyads than between mother-nonmother dyads.
3. The ratio of infants per adult female in an OMU should be inversely related to duration of grooming bouts in which nonmothers groom mothers. Specifically, nonmothers are expected to groom mothers for a longer duration when fewer infants are available.
4. Assuming that nonmothers are more attracted to younger and more dependent infants than to older infants, we expect infant age to be negatively correlated with the amount of grooming received by a mother to obtain access to her infant.
5. The length of grooming bouts provided by nonmothers is expected to be negatively correlated with the rank difference between mothers and handlers. In addition, female handlers that outrank mothers are expected to maintain access to infants for a longer period of time than handlers who are of lower rank than mothers.
6. The amount of time that nonmothers have access to infants is expected to be positively correlated with the amount of grooming they provide that infant’s mother.

## Methods

All research protocols reported in this manuscript were reviewed and approved by the Chinese Academy of Science. This research adhered to the regulations and applicable national laws of the Zhouzhi National Nature Reserve (ZNNR), China, where the study took place, and to the American Society of Primatologists principles for the ethical treatment of primates. This research received clearance from and complied with the protocols approved by animal care committees of the Wildlife Protection Society of Shaanxi Province, China (permit number: SX43537ACC). Legal permission to conduct this research also was provided by ZNNR.

### Study Site

The study site is located in the Yuhuangmiao region of Zhouzhi National Nature Reserve on the northern slopes of the Qinling Mountains, in Shaanxi province, China (108°14′–108°18′E,33°45′–33°50′N). The ZNNR was established in 1985 to protect 52,931 km^2^ of temperate forest and is characterized by a semi-humid montane climate. Elevation ranges from 1,400 to 2,890 m above sea level. The composition of the forest varies with altitude, from deciduous broad-leaf forest at low elevations to mixed coniferous broad-leaf forest above 2,200 m, to coniferous forest above 2,600 m [55]. The terrain is extremely mountainous. During the study period, the average annual temperature was 10.7°C with a maximum of 31.5°C, in July and a minimum of –14.3°C in January. The diet of the golden snub-nosed monkey is characterized by considerable seasonal variation, but consists principally of 29.4% fruit/seeds, 29.0% lichen, 24.0% leaves, 11.1% bark, 4.2% buds, 1.3% twigs and 1.0% unidentified items (based on time spent feeding, [56]).

### Study Troop

Two polygynous groups of golden snub-nosed monkeys are present ZNNR, the east ridge troop (ERT) and the west ridge troop (WRT). The WRT was chosen as the study troop and is characterized by a multi-level social structure consisting of one to two all-male bands and 6–11one-male units (OMU). Details of the study troop have been reported previously [50], [57]. All members were individually recognized by prominent physical characteristics, such as differences in body size, facial features, pelage color, hair patterns, physical disabilities, or the shape of granulomatous flanges on both sides of the upper lip [54].

### Data Collection

Data presented in this study are based on 1,956 hr of behavioral observations collected from March 2009 to 2011 July. Given that individuals of the same one-male unit, especially females, usually remain in close spatial proximity, we were able observe all resident adult OMU females simultaneously. Continuous focal animal behavioral sampling [58] during three hour intervals was used to collect data on all grooming sessions that occurred between females. Data also were recorded on the timing, duration, initiator, and recipient of other social interactions such as approaches, infant handling, threats, fighting, and avoidance. The beginning and end of each grooming bout was recorded to the nearest second, along with the identity of the groomer and the groomee. One-male units were observed from 10∶00 to 16∶00 in an alternating sampling schedule across days to ensure that a relatively equal amount of data were collected on each OMU. Depending on the local conditions of the terrain, we were able to observe the focal OMU from a distance of between 0.5 and 50 m. If members of the target OMU were lost from view for a period of greater than 20 minutes, the next OMU on our sampling schedule was selected for study and observed for a period of 3 hrs. Each observation day we targeted two one-male units for study. Overall, we collected an average of 196 hr of behavioral data on each of the 6 OMUs in our study troop.

During the study period, 26 females in 6 OMUs gave birth. One of these females lost her infant a few days after birth, probably due to natural causes. Ten of these females gave birth both in 2009 and 2011. Five of the 26 females were primiparous and the remaining 21 females were multiparous. Infants under the age of 6 months were selected for study. Infants of this age class possess a distinctive natal coat and are the most attractive to other animals. In total we observed 36 infants during the study period.

Given that we have been observing this troop for over a decade, maternal kinship and non-kin relationships are known for all group members. Approaches were scored when potential handlers or mothers moved to within one arm’s length of the infant. We also recorded the time and social context during periods when handlers or mothers approached or moved away from the infant. A grooming bout was defined as a continuous period of allogrooming lasting at least 10 seconds. A groom bout was scored as ending when either the direction of grooming changed (A groomed B became B groomed A) or when there was a cessation in grooming of more than 60 s. A grooming session is a series of bouts of grooming in either direction between two individuals that was not interrupted for more than 10 min. Infant-handling behaviors recorded included visual inspection, nuzzle, hold, carry, nurse, cradle, groom, and touch (Table 1).

**Table 1:** Definitions of Infant handling behaviors.

Behavior	Definition
Nuzzle	Handler’s nose contacted the infant’s body.
Cradle	Handler supported the infant using one or both arms and brought the infant to her chest.
Carry	Handler maintained ventral-ventral contact with an infant while walking >1 m.
Groom	Handler manipulated an infant’s hair with its hand and/or mouth (except for momentary touching), sometimes removing and eating small items found in the infant’s fur
Touch	Handler gently used its hand to made contact with the infant
Hold	Handler grabbed an infant using one or both hands.

### Data Analysis

To evaluate the effects that the presence of an infant had on female-female social interactions, we calculated hourly rates of approaches and the proportion of time a female received or gave grooming 5 months before and 6 months after giving birth. Two-way repeated measures ANOVAs were used to examine whether there were differences in the rate of approaches or frequency of grooming related to maternal status (mother vs. nonmother) and direction of behavior (given and received ). Individual data were compared using the Paired sample *t*-test to analyze whether females’ were approached more frequently and groomed for longer periods after giving birth than before parturition. The Paired sample *t*-test also was used to analyze whether there was a difference between the proportion of grooming mothers’ received and the proportion of grooming mothers gave on each day. We used a binomial test to determine whether grooming sessions were more likely to be initiated by nonmothers. A *G* test was used to compare the proportion of reciprocal grooming sessions in mother-nonmother dyads and nonmother –nonmother dyads. Grooming sessions directly linked to sexual interactions were excluded. A Wilcoxon signed-ranks test was conducted to analyze whether potential handlers preferentially selected mothers as grooming partners. The observed proportion of grooming episodes received by mothers from potential handlers for each day was compared with the proportion of grooming episodes mothers were expected to receive in relation to their availability (*N* = 197 data collection day).

We used multilevel mixed-effects logistic regressions to determine whether grooming by handlers affect maternal responses to attempts by handlers to interact with infants. Maternal responses were recorded as ‘positive’ when mother allowed a handler to interact with her infant or ‘negative’ when mother left the area or moved her infant away from an approaching handler. Maternal responses were entered as dependent variables and grooming given by potential handlers, the age of the infant (months) at the time the grooming bout was observed, rank difference between potential handler and mother, the current number of newborn infants (≤6 months) per female in each one-male unit were included as independent fixed effect variables. Grooming given by, potential handlers was tested either as a categorical (yes/no occurrence) variable. Data points here were all dyadic interactions between mothers and potential handlers in which the individuals from the initial approach to the final leaving of either the mother or the potential handler in observation (*N* = 897). A *G* test was used to investigate whether handlers in mother–mother dyads were significantly more likely to obtain access to infants without first grooming the mother compared to handlers in mother– nonmother dyads.

To investigate whether the predictions 3–5 (factors affecting the grooming time nonmothers invested in mothers) were supported, multilevel mixed-effects linear regressions was used which examines each of the fixed effects. The duration of grooming (s) given by handlers was used as the dependent variable. For each grooming point, fixed effects consisted of the current number of newborn infants (≤6 months) per female in each one-male unit, the age of the infant (months) when the grooming bout was observed, and the rank difference between the mother and the handler. The position of females in their OMU dominance hierarchy was determined based on the outcome of decided agonistic events between females [59]. Dominance rank was assessed using the dominance index method and used to analyze the direction and the amount of aggressive and submissive behaviors observed [47], [60]. Rank difference was defined as the recipient’s dominance rank subtracted from the initiator’s dominance rank. The rank of the alpha female’s was recorded as ‘1’. Thus, rank difference could range from negative to positive values [13]. To prevent pseudoreplication, the identity of handlers and mothers were inserted as random effects, crossed with each other and nested in the OMU identity. Data points entered in this analysis were dyadic interactions between mothers and potential handlers that the handlers groomed mothers and exchanged for accessing to infants (*N* = 756). Grooming data were standardized by subtracting the mean and dividing by the standard deviation to show whether the duration of grooming in each OMU fluctuated with the number of infants present during each of the three different birth seasons. The mean grooming time for each OMU was transformed by subtracting mean grooming bout length from the length of each grooming bout and dividing by the standard deviation in each of the 3 birth seasons over which this study was conducted. A multilevel mixed-effects linear regression was also used to investigate whether high-ranking potential handlers were better able to get access to younger or relatively less infants. The rank difference between the mother and the handler were entered as the dependent variable, and age of infants and the number of infants per female were included as independent fixed-effect variables. The identity of handlers and mothers were inserted as random effects, crossed with each other and nested in the OMU identity. Data points were the number of that the handlers got accessing to infants from mothers (*N* = 781). To test whether predictions 5 and 6 were supported (impact of grooming time and rank difference on infant handling time), we also used multilevel mixed-effects linear regressions. The duration of infant handling (s) were defined as the dependent variable. Fixed effects included the duration of the handler’s grooming bout, the current number of infants (≤6 months) per female in each OMU, the age of the infant when the grooming bout occurred, and the rank difference between the mother and the handler. The results showed that rank difference did not have a significant effect on handling time. However, we noted that certain females in each OMU were permitted to handle infants for longer periods of time when rank difference interacted with infant’s age and the number of infants presented in an OMU. In addition, previous analyses performed on grooming sessions outside birth and mating seasons revealed that even if females had as many as five partners to select from, most grooming occurred between two closely ranked females who interacted as frequent groomers [13]. Using criteria outlined in Barrett et al. (2000) [61], if individuals in the same dyad spent more than 5% of their total active and passive grooming time grooming, they were defined as frequent partners. Considering this, the effect ‘frequent groomer’ was used instead of ‘rank difference’ to test the prediction that female handlers who were frequent grooming partners maintained access to infants for a longer period of time than handlers who were infrequent partners. A dichotomous variable was used to represent mother and handler dyads for all handling times: ‘positive’ when the females were frequent grooming partners outside the birth and mating seasons, and ‘negative’ when females were not frequent grooming partners outside of the birth and mating seasons. In order to prevent distortion of the results due to pseudo-replication, random effects included in this analysis were the identity of the handlers and mothers, crossed with each other and nested in the OMU identity. Dyadic interactions as data points entered here were between mothers and potential handlers in which the potential handler had access to the infant (*N* = 756). Data were analyzed using the SAS 9.2 statistical package.

## Results

**Figure 1:**
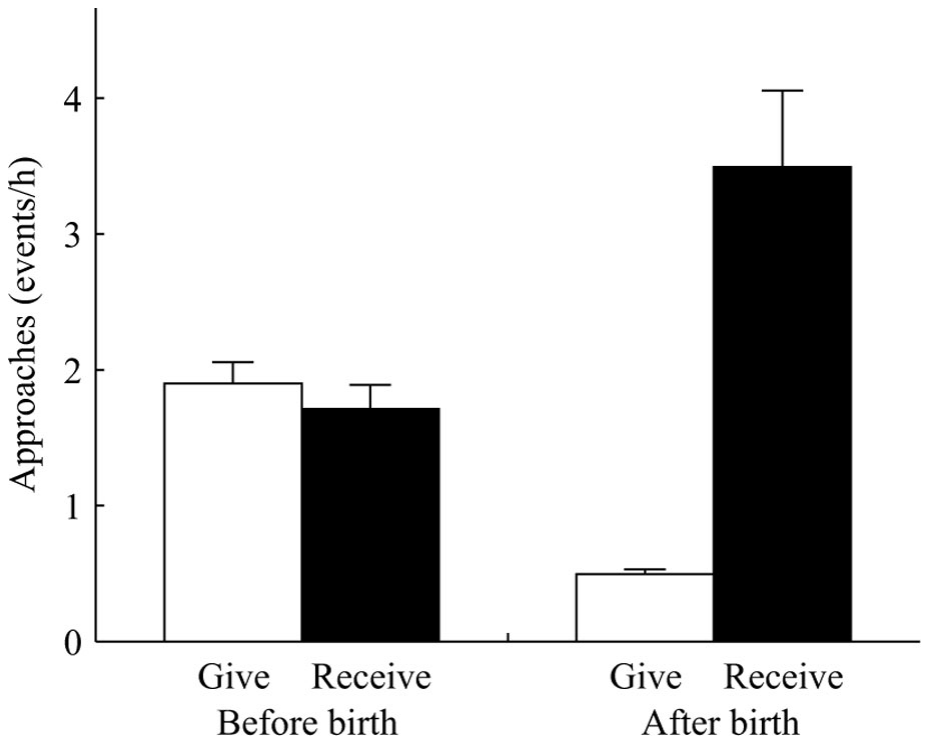
The ratio of approaches per hour (mean ± SE) mothers gave (▪) and received (□) before parturition and after giving birth. Mother received significantly more approaches from nonmothers in the months following infant births than in the 5 months prior to giving birth (paired *t* test: *t*_35_ = −10.01, *p*<0.05). New mothers approached other females less after giving birth than before parturition (paired *t* test: *t*_35_ = 12.05, *p*<0.05).

The presence of infants significantly influenced the rate at which handlers approached mothers. We found that the rate at which mothers or nonmothers were approached differed significantly (*F*_1,70_ = 23.17, *p*<0.05; Fig. 1). Based on our sample (N = 897) potential handlers were responsible for 81.7% of total approaches in nonmother-mother dyads. Females were approached at a significantly higher rate by nonmothers in the 6 months after they gave birth (paired *t* test: *t*_35_ = −10.01, *p*<0.05) compared to the 5 months prior to giving birth (paired *t* test: *t*_35_ = 12.05, *p*<0.05). In contrast, nonmothers were approached less frequently than mothers (paired *t* test: *t*_47_ = −25.31, *p*<0.001), and the rate of approaches among nonmother-nonmother dyads decreased during the birth season compared to the period prior to infant births (paired *t* test: *t*_47_ = 15.11, *p*<0.05). During the 6 month period after mothers’ gave birth, the rate nonmothers approached mothers was significantly higher than the rate at which they approached nonmothers (paired *t* test: *t*_47_ = −14.17, *p*<0.001).

**Figure 2:**
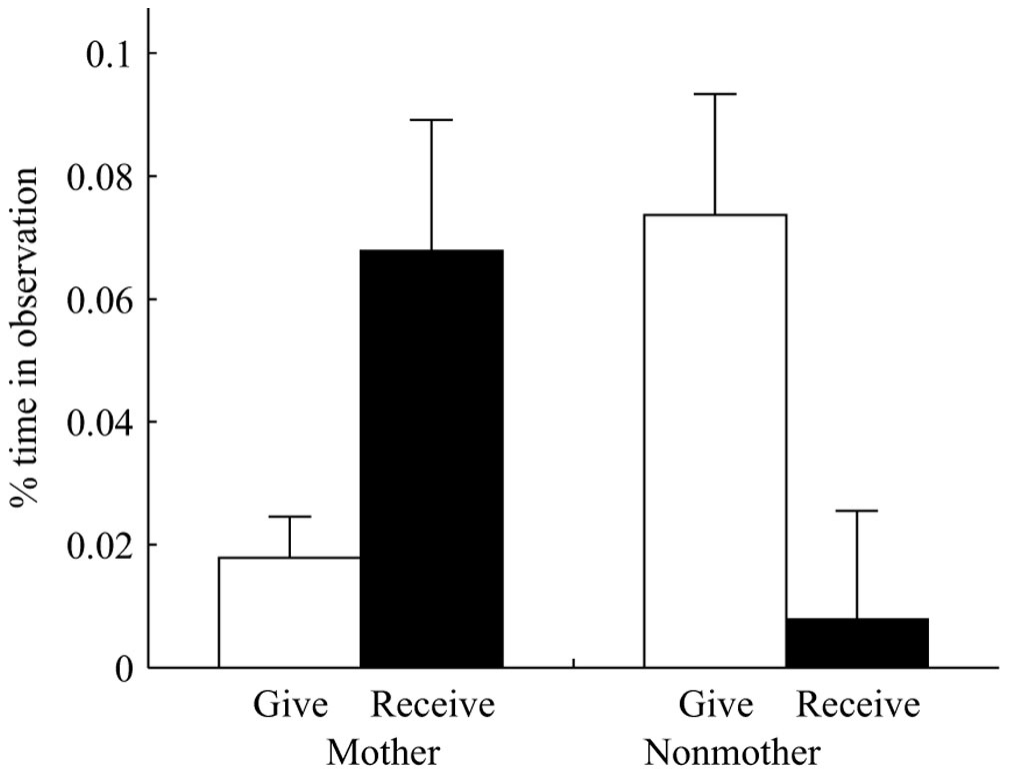
Percentage of time grooming (mean ± SE) mothers gave (▪) and received (□) compared to the grooming nonmothers gave and received. The proportion of grooming mothers received from nonmothers was significantly higher than the proportion of grooming mothers gave to nonmothers on each day after parturition (paired *t* test: *t*_35_ = −9.43, *p*<0.01). Nonmothers gave significantly more grooming to mothers than they received from mothers (paired *t* test: *t*_47_ = −13.72, *p*<0.01).

Grooming also was significantly affected by the presence of infants (*F*_1,70_ = 37.12, *p*<0.01). A total of 756 grooming-infant handling interchanges were recorded between mothers and nonmothers. Of these, 89.3% were initiated by potential handlers and only 10.7% initiated by mothers. We found that grooming sessions were more likely to be initiated by nonmothers (binomial test: *N = *756, observed *P = *0.89, theoretical *P = *0.5, *p*<0.001) and less likely to be reciprocated by mothers (*G*_1_ = 901.73 *p*<0.001). Mothers were chosen as grooming partners significantly more than expected based on their relative presence within their OMU (*Z* = −8.41, *N* = 197, *P*<0.05). An analysis of grooming sessions indicated that 83% were unidirectional and mothers reciprocated grooming on only 17% of occasions. In contrast, 91.7% of the total 673 grooming session between nonmother dyads were bidirectional and nonmothers commonly reciprocated by grooming their partners. The average duration of grooming bouts in which nonmothers groomed mothers was 241.4±292.8 s (mean ± SD). Mothers were groomed by nonmothers for a longer duration after they gave birth (241.4±292.8 s) than before parturition (197.1±121.7 s) (paired *t* test: *t*_35_ = −8.521, *p*<0.001). However the proportion of time mothers spent grooming others decreased across these two periods (paired *t* test: *t*_35_ = 11.41, *p*<0.05) while the proportion of time nonmothers spent grooming mothers increased (paired *t* test: *t*_47_ = −19.21, *p*<0.05). Similarly, the proportion of grooming mothers with young infants received from nonmothers was significantly higher than the proportion of grooming these mothers directed at nonmothers (paired *t* test: *t*_35_ = −9.43, *p*<0.001). Similarly, nonmothers groomed mothers for a significantly longer period of time than they were groomed by mothers (paired *t* test: *t*_47_ = −13.72, *p*<0.05; Fig. 2), and the proportion of total grooming time nonmothers invested in mothers was significantly higher than the proportion of total grooming time they invested in nonmothers (paired *t* test: *t*_ 47_ = −12.63, *p*<0.05). Finally, nonmothers groomed mothers for significantly longer periods of time than they grooming other females (paired *t* test: *t*_ 47_ = −3.01, *p*<0.05).

Whether nonmothers groomed mothers prior to handling infants has significant effect on maternal responses to attempts by nonmothers to interact with infants. Grooming by nonmothers prior to handling infants increased the probability of nonmothers accessing to the infants (Table 2) and decreased the probability of nonmothers either being threatened or chased away by mothers (Table 3). When grooming preceded handling, mothers were more likely to sit passively and allow nonmothers to handle their infant (93%, 756/813). In cases in which nonmothers attempted to handle infants without first grooming the mother, mothers commonly threatened or moved away from the potential handler (90%, 223/248). The longer nonmothers groomed, the more likely they were allowed to remain in close proximity to mothers and handle their infants (*Z = *1.49, *N = *756, *P = *0.012).

**Table 2:** Probability of positive maternal responses in relation to grooming given by potential handler.

Independent variables	Coefficient	Z	*p*
Grooming given	0.72	3.91	0.001
Number of infants	−0.13	−0.49	0.126
Age of infants	0.38	0.74	0.301
Rank difference	0.25	0.66	0.249

Positive maternal responses – mother allowed a handler to interact with her infant. Other independent variables are the number of infants per OMU female, the age of the infant and the rank difference between the mother and the handler. *N = *897.

**Table 3:** Probability of in relation to grooming given by potential handlers.

Independent variables	Coefficient	Z	*p*
Grooming given	−0.93	−3.79	0.017
Number of infants	−0.32	−1.61	0.138
Age of infants	0.28	0.67	0.274
Rank difference	0.58	1.34	0.103

Negative maternal responses – mother left the area or moved her infant away from an approaching handler. Other independent variables are the number of infants per OMU female, the age of the infant and the rank difference between the mother and the handler. *N = *897.

We recorded only 29 cases infant handling that occurred across mother-mother dyads. The duration of mother-mother grooming was shorter (112.2±79.3 s vs. 241.4±292.8 s) than nonmother-mother grooming (*t*_257_ = −18.94, *p*<0.001). Handlers in mother–mother dyads were significantly more likely to obtain access to infants without first grooming the mother than were handlers in mother– nonmother dyads (mother–mother: with grooming: 20.7%, *N = *6; without grooming: 79.3%, *N = *23; mother– nonmother: with grooming: 92.4%, *N = *699; without grooming: 7.6%, *N = *57; *G*_1_ = 83.09, *p*<0.001).

**Figure 3:**
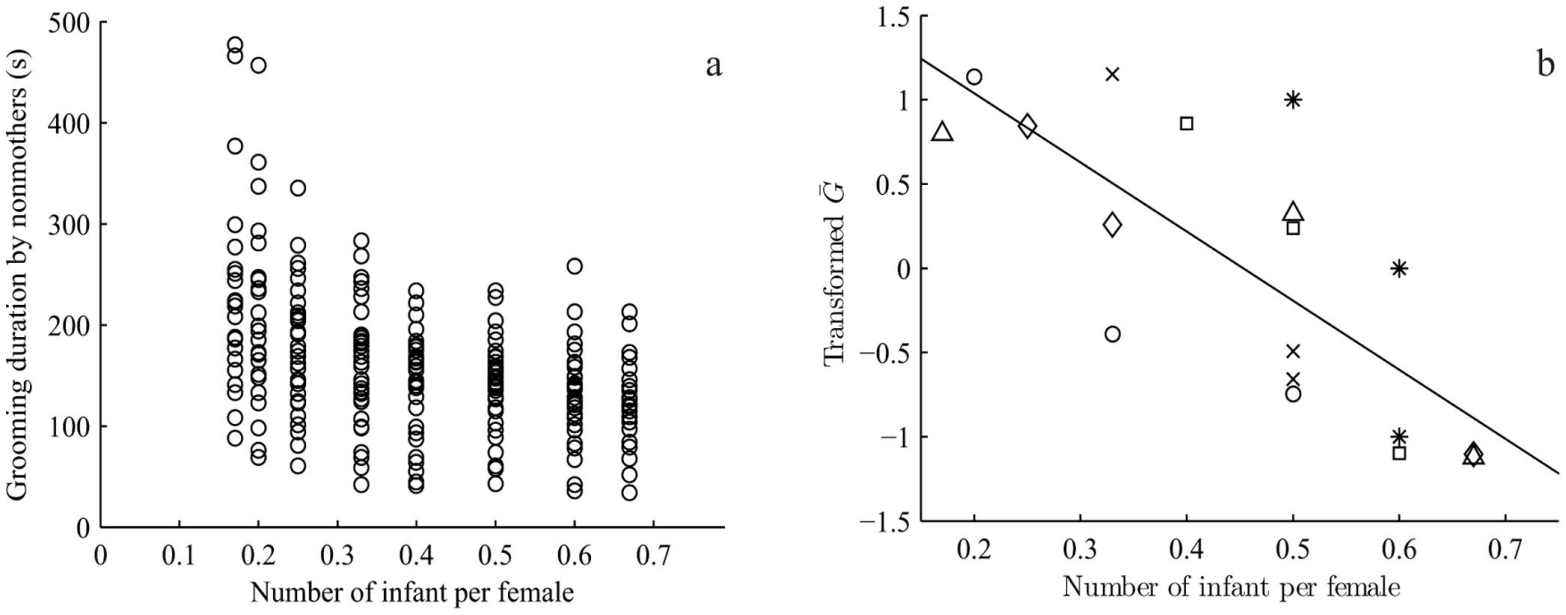
(a) The duration of grooming nonmothers invested in mothers before getting access to the infants in relation to the number of infants per female in each one-male unit. When the number of infants per female in each one-male unit increased the amount of grooming given to mothers also decreased. (b) The duration of grooming varied with the number of infants per female during each of the 3 infant birth seasons in a given OMU (total 6 OMUs). Similarly the amount of grooming that nonmothers gave to mothers was negatively correlated with the number of infants in the OMU. The 6 OMUs are identified as follows: JB, FP, PK, JZT, BB, RX. Each sign represents one OMU in one birth season–JB (○) FP (*) PK (□) JZT (◊) BB (Δ) RX (×).

**Figure 4:**
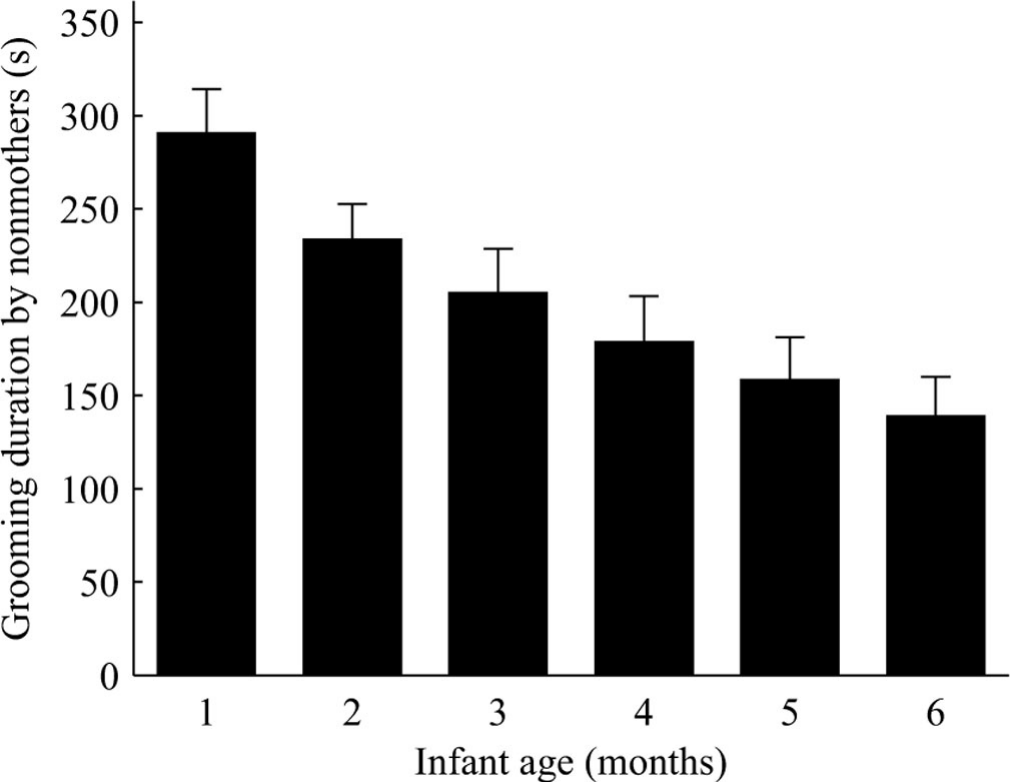
The amount of grooming that nonmothers (mean ± SE) gave to mothers was negatively correlated with infant age.

The number of infants per female, age of infants, and rank difference had a significant impact on the amount of time nonmothers invested in grooming mothers. Grooming duration increased significantly when the number of available infants per female in each OMU decreased (Coefficient = −0.45, *Z* = −2.12, *N = *756, *P*<0.001, Fig. 3a). The grooming nonmothers gave to mothers before accessing to infants was significantly affected by the current supply of infants in each OMU. Moreover, within a single OMU the duration of grooming varied consistently with the number of infants per female across three breeding periods (Fig. 3b). In each case, as the number of infants per female decreased, the longer handlers were found to groom mothers. When the number of infants per OMU increased, grooming durations were shorter. The infants’ age was associated with a decreased duration of grooming nonmothers gave to mothers (Coefficient = −0.11, *Z* = −1.20, *N = *756, *P* = 0.021, Fig. 4). The duration of grooming decreased significantly as infants grew older. Handling older infants required less grooming over shorter durations. Similarly, there was evidence that increasing rank difference negatively influenced grooming duration of nonmother-to-mother grooming bouts that was required before handlers were able to touch or groom infants (Coefficient = −0.57, *Z* = −0.94, *N = *756, *P* = 0.017). The higher ranking the handler was compared to the mother, the less time was invested in grooming the mother. The greater the rank difference between a lower ranked female and a mother, the greater the duration of time that female groomed the mother in order to obtain access to her infant. In addition, the rank difference between nonmothers and mothers was significantly related to the age of the infant (Coefficient = 0.67, *Z* = 3.18, *N = *781, *P* = 0.015, controlling for the number of infants per female). When infants were young, mothers and handlers were closely ranked or handlers were of higher rank than mothers, supporting the notion that access to young infants was a valued commodity sought after by nonmothers. However, nonmothers of lower rank than mothers were not excluded from getting access to infants when the number of infants per female was relatively low in the OMUs (Coefficient = −0.98, *Z* = 2.12, *N = *781, *P* = 0.157, controlling for the age of infant).

The number of infants per female significantly predicted the duration of infant handling. The duration of infant handling decreased significantly when the number of available infants increased (Coefficient = −0.27, *Z* = −1.61, *N = *756, *P* = 0.011). Grooming times only had a significant influence on infant handling time when controlling for the number of infants per female in each OMU (Coefficient = 0.66, *Z* = 2.39, *N = *756, *P* = 0.031). Nonmothers who groomed mothers longer increased their access to infants only when infant availability also increased. Frequent female grooming partners significantly affected the infant handling duration (Coefficient = 0.76, *Z* = 2.19, *N = *756, *P*<0.001). Frequent groomers were allowed to handle infants significantly longer than infrequent grooming partners and the duration of infant handling decreased significantly when females were not frequent groomers. The duration of infant handling was not significantly affected by the infant age (Coefficient = 0.58, *Z* = 1.95, *N = *756, *P* = 0.812 ).

## Discussion

The biological market model predicts that trading decisions between partners will be influenced by the current ‘exchange rate’ of particular commodities which is a function of each partners’ need for the commodity and the ability to outbid or outcompete other group members who also can provide a needed good or service [1], [2].Thus, the ‘price’ of a commodity is expected to fluctuate with supply and demand. In many species of primates, mothers and their infants represent a central focus of interest for females in the group [30], [31], [62], [63], [64]. Mothers are generally tolerant of approaches that result in nonmothers being in close spatial proximity to their offspring. However, direct physical access to offspring requires that nonmothers invest time and effort in first grooming the infant’s mother.

The results of this study indicate that the presence of newborn infants has a significant effect on the dynamics of adult female social interactions in wild golden snub-nosed monkeys (*Rhinopithecus roxellana*). Females received more approaches and were groomed for longer periods of time in the months following the birth of their infant than in the months prior to parturition. Females without infants (nonmothers) were strongly attracted to newborn infants and preferentially groomed mothers as a social tool to obtain access to their infants. Nonmothers were found to groom mothers more frequently and for a greater duration than mothers groomed them in return. Previous research on *R*. roxellana [13] indicated asymmetries in female grooming relationships with factors such as rank and the number of available grooming partners having a significant effect on the degree to which grooming was reciprocated. In the present study, grooming an infant’s mother increased the potential handlers’ chances of establishing contact with the infant. Given that grooming initiated by nonmothers was rarely reciprocated by the infant’s mother, our results support the contention that in golden snub-nosed monkeys females without infants interchange grooming for access to infants (Prediction 1 supported). In contrast, we found that grooming between mother-mother dyads was reciprocal, and occurred significantly less frequently than grooming between mother-nonmother dyads (Prediction 2 supported). Thus, it appears that individual females adjust their social strategies and grooming relationships depending on several factors including their reproductive status, availability of young infants, and position in the dominance hierarchy. For example, the duration of grooming bouts that nonmothers directed toward mothers increased significantly when fewer infants were present in an OMU, and with an increase in rank difference between the mother and infant handlers (Prediction 3 supported and Prediction 5 partly supported). In contrast, the duration of grooming traded for access to infants decreased significantly with infant age (Prediction 4 supported). The fewer infants present, the greater amount of time handlers groomed mothers for access to their infant. Such evidence implies that similar amounts of grooming had a decreasing effect on the probability of getting access to infants when the number of infants per female in the OMUs decreased. Our findings suggest that in golden snub-nosed monkeys the grooming of mothers by nonmothers is a service that is actively interchanged for infant handling privileges, and that the ‘value’ of infants changed depending on supply and demand in the biological market place of the *R. roxellana* OMU. These results support the biological market prediction that the scarcity of a resource or a service increases its value, while the increased availability of that resource or service decreases its value [3]. Studies of several other primate species also have demonstrated that the relative number of infants present in a group can influence the duration of grooming received by mothers who interchanged grooming for access to infants [26], [41], [43], [45].

Allowing others access to her offspring is likely to be a source of anxiety for the mother especially during the infants first few months of life [11], which is a period of infant vulnerability [40]. Moreover, inexperienced females may mishandle infants, carry infants away from the mother, or continue to retain access to infants during periods when they need to nurse. It has been argued that given the important role that grooming plays in reducing stress, affiliative social contact with the mother prior to attempting to handle her infant may serve to reduce maternal anxiety and facilitate the groomer’s access to the infant [28], [41], [65]. This appears to be an important factor affecting the interchange of grooming for infants contact in chacma baboons [41]. However, factors in addition to supply and demand ratios, may affect the ‘value’ of handling infants. For example, we found an effect of infant age on the interchange of grooming for infant access. Nonmothers groomed mothers longer for access to infants less than 6 months of age than for access to infants older than 6 months of age. What remains less clear is whether this is the result of a decrease in the attractiveness of older infants to handlers (supply of infants remains the same but handler demand/motivation decreased) or whether mothers of older infants are less anxious or more permissive and allow others to handle their offspring (supply of infants remains the same but cost required by mothers for access to infants has decreased) or whether the main goal of handling is to get to know the new group member and form a (positive) relationship with this individual and this becomes less necessary when the infant grows older.

We also found an effect of female rank on patterns of grooming associated with access to infants. In golden snub-nosed monkeys, rank appears to shift the balance for many individuals from dyadic interactions based on reciprocity to dyadic interactions that are characterized by the unequal exchange of the same service or the interchange of differences services. For example, lower-ranked females groomed higher-ranked mothers for longer duration than vice versa. On average higher-ranking females grooming lower ranking mothers for 213.7±171.3 seconds (mean ± SD) whereas lower-ranking females groom higher ranking mothers an average of 261.2±234.1 seconds in order to obtain access to their infants (Prediction 5 partly supported). This suggests that lower-ranked and higher-ranked females are characterized by different social strategies and these change with infant attractiveness. Higher-ranked nonmothers were able to ‘pay a lower price’ than the one set by the current supply/demand ratio or paid back in a different ‘currency’, e.g. tolerance at food sources or support in conflicts. When infants were very young, handlers were more likely to be closely ranked or of higher rank than the infant’s mother. However, this pattern did not continue once infants reached 6 months of age. This suggests that when infants were most highly valued, higher-ranked nonmothers successfully outbid lower-ranked competitors for infant access. As these infants aged, however, their ‘value’ appeared to decrease and lower-ranked handlers had greater access to infants by increasing the time they invested in grooming higher-ranking mothers. If access to higher-ranked mothers and their infants are of greater value in the biological market place than access to lower-ranked females and their infants, then the primary goal of lower ranking females may have been to use access to infants as a means of developing a social relationship with a female of higher rank. These same low-ranking females could gain equal infant caregiving experience at a lower cost (reduced time grooming) by preferentially grooming mothers of low social rank. In contrast, it is likely that higher ranked females who groom lower ranking mothers do so for the primary purpose of obtaining infant caregiving experience, as social bonds or alliances with these low ranking mothers offer limited benefits. This may be analogous to data obtained in studies of captive female tamarins (*Saguinus*) and marmosets (*Callithrix*) [17]. In these primates experience that young females obtain in caring for another’s offspring is argued to be a critical factor increasing the survivor ship of that female’s future offspring [66]. Taken together, our results indicate that the market value of infants depends on multiple factors, some of which are generally stable over the birth season (OMU size and composition, female social rank, experience caring for offspring) and others that may fluctuate based on changes in outbidding competition, infant age, and supply and demand ratios.

Finally, several previous studies [41], [42], [43], [67] have focused on infants as a tradable commodity with their value represented by the costs incurred (grooming time and energy) by nonmothers. Less attention has been paid to the quality of the infant handling experience obtained by nonmothers and its potential benefits to nonmothers in alliance formation, change in dominance status, and future reproductive success [45]. In order to best understand the social dynamics of the biological market place greater emphasis needs to be laid on understanding and better estimating variation in the cost and value to individual traders (mothers and handlers) of access to an infant and access to that infant’s mother. In the present study, the duration of grooming did not directly correlate with infant handling times, and thus Prediction 6 was not supported. Only when the number of available infants increased, did an increase in the duration of a grooming bout result in increased access to infants. When the number of infants in an OMU was limited, longer grooming bouts did not necessarily result in increased handling time. The results also revealed that when the number of available infants increased, handling time decreased. When females and mothers were infrequent grooming partners, the duration of infant handling also decreased. However, nonmother frequent grooming partners spent a significantly greater amount of time handling infants than did nonmother infrequent groomers regardless of the number of infants present in each OMU. This suggests that persistent or long-term social partners are characterized by a preferred or special relationship that may enable them to receive a discount in the biological market place. Female *R. roxellana* commonly form alliances in the context of grooming frequently, moving, feeding, stay and even fighting together, and thus long-term social partnerships (whether kin or nonkin based) offer advantages in fitness (Wei Wei, unpublished data).

Our results suggest that in *R. roxellana* access to infants represents a commodity that can be traded for grooming. The ‘value’ of infant handling varied based on supply and demand, the social status of the individual traders, and whether potential handlers had a long-term relationship with the infant’s mother. Given that golden snub-nosed monkeys are strictly seasonal breeders, more than one similarly aged infant is commonly present in an OMU. Nevertheless nonmothers from one OMU do not groom mothers from neighboring OMUs or interact with their infants. Therefore, the availability of infants in a female’s resident OMU has a strong impact on female social interactions. The presence of young infants appears to have a direct influence on female partner choice, grooming patterns, and the formation of long-term alliances. Mother’s with newly born infants were groomed for a longer period of time than nonmothers or mothers with older offspring. Differences in the amount of grooming females traded for access to another female’s infant appeared to be based on market ‘rules’ of supply and demand as well as female rank. Although in the case of higher ranking females, reciprocity or long-term alliances may help explain the value or costs of exchanging goods and serves, for many other females directly responding to the current value of goods and services may reduce the need for complex calculations, cost-benefit analyses or bookkeeping. We plan to continue our research on social interactions and interchange trading in golden snub-nosed monkeys in order to better identify how individuals adjust their social behavior in the face of short-term changes in the value of goods and services.
